# Normal glycosylated hemoglobin masking glucose dysregulation in a patient with pancreatic and hematologic disease

**DOI:** 10.1210/jcemcr/luag037

**Published:** 2026-03-13

**Authors:** Jean Carlos Ramos Cardona, Jacqueline Rodriguez Gilmore, Tathana Rivera Hernandez, Suzanne Quinn Martinez

**Affiliations:** Department of Internal Medicine, HCA Florida Orange Park Hospital, Orange Park, FL 32073, USA; Department of Internal Medicine, HCA Florida Orange Park Hospital, Orange Park, FL 32073, USA; Department of Internal Medicine, HCA Florida Orange Park Hospital, Orange Park, FL 32073, USA; Department of Internal Medicine, HCA Florida Orange Park Hospital, Orange Park, FL 32073, USA

**Keywords:** glycemic instability, fructosamine, clonal hematopoiesis of indeterminate potential, pancreatic cystic lesions, intraductal papillary mucinous neoplasm, pancreatic neuroendocrine tumor

## Abstract

Although hemoglobin A1c (HbA1c) is widely used to assess long-term glycemia, its reliability declines in conditions that alter red blood cell turnover or hemoglobin glycation. Pancreatic structural diseases, including pancreatic neuroendocrine tumors (pNETs) and intraductal papillary mucinous neoplasms (IPMNs), may further affect glucose regulation through impaired endocrine function. Systemic inflammation and specific hematologic conditions can also create discordant glycemic markers, complicating diagnosis, and management. We report a 59-year-old woman with autoimmune disease who presented with fatigue and fluctuating glucose levels. Her HbA1c remained within normal limits; however, continuous glucose monitoring (CGM) and an oral glucose tolerance test (OGTT) demonstrated marked hyperglycemia. Imaging revealed pancreatic lesions concerning for a pNET in the setting of known IPMNs. Laboratory evaluation was notable for elevated ferritin and clonal hematopoiesis of indeterminate potential (CHIP). Additional studies, including hemoglobin, albumin, renal and hepatic function, and hemoglobin electrophoresis, were normal, ruling out anemia and hemoglobinopathies. Her glycemic discordance likely reflects impaired insulin secretion due to pancreatic pathology combined with inflammation driven alterations in erythrocyte lifespan associated with CHIP. This case underscores the limitations of HbA1c in complex metabolic or inflammatory states and highlights the value of CGM and OGTT when A1c does not align with clinical findings.

## Introduction

When evaluating a patient for diabetes mellitus is typically guided by established markers such as hemoglobin A1c (HbA1c) of greater than or equal to 6.5%, fasting blood glucose of greater than or equal to 126 mg/dL (SI: 7.0 mmol/L) (reference range, 70-140 mg/dL [SI: 3.9-7.8 mmol/L]), 2-hour blood glucose of greater than or equal to 200 mg/dL (SI: >11.1 mmol/L) in oral glucose tolerance test (OGTT) or random glucose greater than 200 mg/dL (SI: >11.1 mmol/L) with classic clinical symptoms of hyperglycemia in a repeated test [1, 2]. However, on rare occasions discrepancies between clinical symptoms, glucose measurements, and HbA1c do not align with the patient's presentation, which can delay diagnosis and complicate management.

Certain factors can result in falsely decreased HbA1c such as red blood cell turnover, decreased red blood cell lifespan, hemoglobin variants, pregnancy, medications, red blood cell transfusions or supplements such as vitamin C and E [3]. Chronic conditions such as chronic kidney disease and advanced liver disease lead to falsely low HbA1c due to altered erythropoiesis and increased erythrocyte turnover [4].

Additionally, clonal hematopoietic conditions have been increasingly recognized as a driver of age-related inflammatory and metabolic dysfunction [5]. Clonal hematopoiesis of indeterminate potential (CHIP) pathogenic variants, particularly in genes associated with coronary heart disease such as *TET2*, have been linked to a 23% increased risk of developing type 2 diabetes (T2D), possibly via atherosclerosis related pathways and insulin resistance [5, 6]. These overlapping mechanisms complicate interpretation of glycemic markers such as HbA1c. In such scenarios, alternative metrics like fructosamine or continuous glucose monitoring (CGM) may offer a more reliable assessment of glycemic status [7].

The aim of this report is to present a diagnostically challenging case in which pancreatic pathology, autoimmune disease, and hematologic factors led to incongruent glycemic markers, underscoring the need to consider complementary testing when standard measurements do not align.

## Case presentation

A 59-year-old woman presented to the endocrinology clinic for evaluation of hyperglycemia. Her medical history was notable for diabetes mellitus, rheumatoid arthritis (previously treated with hydroxychloroquine and prednisone), chronic obstructive pulmonary disease, gastroesophageal reflux disease, and pancreatic cysts. She reported adhering to a strict ketogenic diet for over a year, yet experienced persistent blood glucose levels in the 150 mg/dL (SI: 8.3 mmol/L) with postprandial spikes exceeding 200 mg/dL (SI: 11.1 mmol/L). She also had a thyroid nodule and elevated ferritin for over 15 years, though *HFE* pathogenic variant testing was negative, and magnetic resonance imaging showed no hepatic iron overload.

Her hematologic history included prior identification of a DNA methyltransferase 3A pathogenic variant consistent with CHIP. She had no cytopenias or active hematologic disease, and recent imaging showed resolution of prior splenomegaly.

## Diagnostic assessment

**Figure 1:**
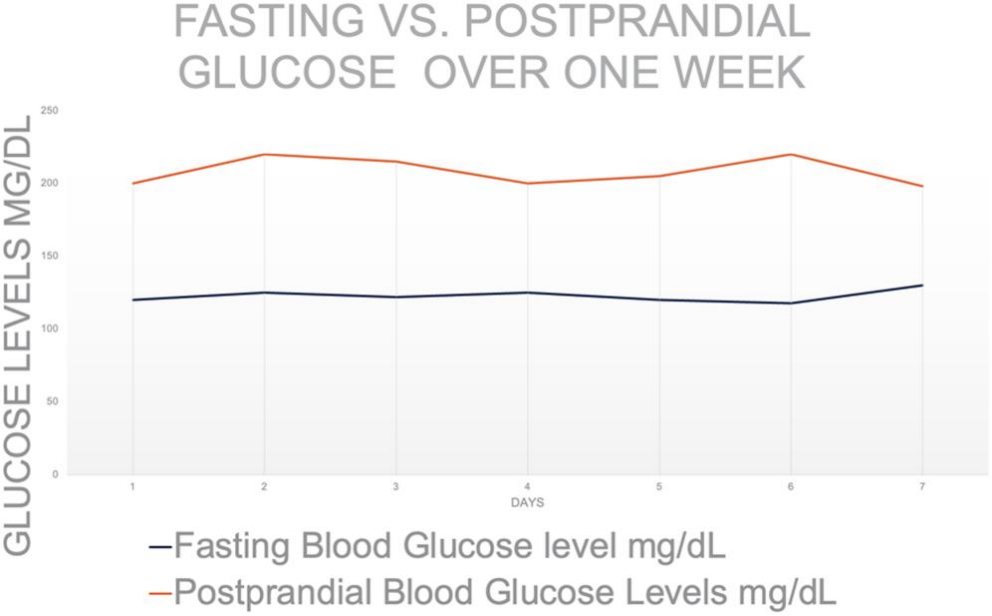
Fasting vs postprandial glucose over 1 week. This line graph illustrates daily fasting blood glucose levels and postprandial (after-meal) blood glucose levels measured in milligrams per deciliter (mg/dL) over the course of 1 week. Fasting blood glucose remained with subtle elevations, while postprandial levels were consistently elevated above 200 mg/dL, indicating significant postprandial glycemic variability. Abbreviation: mg/dL, milligrams per deciliter.

The patient's initial glucose was 141 mg/dL (SI: 7.8 mmol/L) while on empagliflozin, which she felt was ineffective. Medication reconciliation identified no hyperglycemia inducing agents, and the patient was not on corticosteroid therapy for her autoimmune disease. Comprehensive laboratory evaluation included HbA1c, fructosamine, insulin levels, hemoglobin electrophoresis, and iron studies. She was advised to temporarily increase carbohydrate intake for better glycemic assessment. CGM revealed significant postprandial hyperglycemia (Fig. 1), and HbA1c remained at 4.7% (reference range, less than 6.5%). Fructosamine was 265 µmol/L (reference range, 200-285 µmol/L), and insulin was 9 µIU/mL (SI: 62.5 pmol/L) (reference range, 2-19 µIU/mL, [SI: <174 pmol/L]) (Table 1). An OGTT was consistent with hyperglycemia (Table 2).

**Table 1:** Summary of longitudinal glycemic and related laboratory markers (2020-2024)

Test	Reference range	2020	2021	2023	2024	2025
HbA1c (%)	< 5.7%	4.9%	4.9%	5.0%	4.7%	5.2%
Fructosamine	200-285 µmol/L	ND	276 µmol/L	303 µmol/L	265 µmol/L	ND
GlycoMark	≥10 µg/mL normal	ND	4.0 µg/mL	ND	ND	ND
Hemoglobin	11.1-15.9 g/dL (SI: 6.9-9.9 mmol/L)	ND	13.9 g/dL (8.6 mmol/L)	ND	14.5 g/dL (9.0 mmol/L)	ND
Albumin	3.5-5.2 g/dL (SI: 35-52 g/L)	ND	ND	4.0 g/dL (40 g/L)	4.5 g/dL (45 g/L)	ND
Insulin	2-25 µIU/mL (SI: 14-174 pmol/L)	ND	ND	18.6 µIU/mL (129 pmol/L)	9 µIU/mL (62.5 pmol/L)	ND
GMI (14 days)	< 5.7%	ND	ND	ND	ND	7.9%

Abbreviations: µIU/mL, micro-international units per milliliter; µmol/L, micromoles per liter; GMI, glucose management indicator, estimates your average A1C level using data from a continuous glucose monitor (CGM); HbA1c, hemoglobin A1c; pmol/L, picomoles per liter; pmol/L, picomoles per liter.

This table summarizes the patient's glycemic biomarkers over a 4-year period, including HbA1c, fructosamine, GlycoMark, hemoglobin, albumin, and fasting insulin. The table highlights persistent discordance between HbA1c and alternative glycemic markers, particularly fructosamine despite consistently normal hemoglobin and albumin levels in the setting of hyperglycemia.

**Table 2:** Oral glucose tolerance test (OGTT) demonstrating markedly impaired glucose regulation

Test	Value	Reference value
Glucose, fasting	198 mg/dL (SI: 11.0 mmol/L)	70-99 mg/dL (SI: 3.9-5.5 mmol/L)
Glucose ½ hour	355 mg/dL (SI: 19.7 mmol/L)	70-199 mg/dL (SI: <11.1 mmol/L)
Glucose 1 hour	384 mg/dL (SI: 21.3 mmol/L)	70-199 mg/dL (SI: <11.1 mmol/L)
Glucose 2 hours	348 mg/dL (SI:19.3 mmol/L)	70-139 mg/dL (SI: <7.8 mmol/L)

Abbreviations: mg/dL, milligrams per deciliter; mmol/L, millimoles per liter; OGTT, oral glucose tolerance test.

The patient's OGTT shows significantly elevated fasting, 30-minute, 1-hour, and 2-hour plasma glucose concentrations far exceeding diagnostic thresholds for diabetes mellitus.

Hemoglobin electrophoresis ruled out variants: Hgb A 97%, Hgb A2 2.4%, Hgb F 0%, no Hgb S. Protein electrophoresis and *JAK2* pathogenic variant testing were also unremarkable. Imaging with a gallium-68 dodecane tetraacetic acid tyrosine-3-octreotate positron emission tomography (Ga 98-DOTATATE PET) revealed tracer uptake in pancreatic head and tail, correlating with cystic lesions. The magnetic resonance cholangiopancreatography showed multiple pancreatic cysts, the largest being 13 mm in the uncinate process, consistent with serous cystic neoplasm or IPMN. Surgical consultation favored surveillance over intervention, as lesions appeared nonfunctioning and indolent.

## Treatment

Antidiabetic medications were temporarily held to allow physiologic assessment of her endogenous glucose regulation, given the marked discordance between CGM findings and her normal HbA1c. Metformin had previously been discontinued due to intolerance, and semaglutide was avoided because of underlying pancreatic cystic lesions. An OGTT was performed off therapy to characterize the degree and pattern of dysglycemia without pharmacologic influence. After confirming significant hyperglycemia despite a low HbA1c, empagliflozin was resumed as monotherapy during the evaluation period.

## Outcome and follow-up

Follow-up CGM showed an average glucose of 198 mg/dL (11.0 mmol/L) with a nadir of 120 mg/dL (6.7 mmol/L). She also reported a prior asymptomatic glucose low of 65 mg/dL (3.6 mmol/L) while on empagliflozin. Based on these findings, she continued empagliflozin with close monitoring and ongoing dietary management. Her pancreatic lesions remain under annual surveillance without radiographic progression. Ferritin levels decreased following dietary modification, and hematologic follow-up for CHIP continues without evidence of disease evolution.

## Discussion

This case underscores the complexity of diagnosing and managing hyperglycemia in patients with overlapping pancreatic, hematologic, and inflammatory conditions. The discordance between hyperglycemia and normal HbA1c highlighted the importance of adjunctive markers like fructosamine and CGM. Hemoglobin fractionation ruled out variant hemoglobin, while iron studies pointed to chronic inflammation, rather than iron overload, as a potential contributor to altered erythrocyte turnover and falsely low HbA1c. Comprehensive monitoring of blood glucose will allow clinicians to have a better idea of how well blood glucose is being controlled, rather than HbA1c in patients whose HbA1c is falsely low. A normal HbA1c, but elevated fasting blood glucose or elevated blood glucose are still part of criteria for diabetes mellitus.

The coexistence of pancreatic cystic lesions and low-grade tracer uptake on Ga 98-DOTATATE PET imaging raised concern for a pNET; however, the clinical presentation was more consistent with a nonfunctioning, indolent process, such as an IPMN.

Diabetes mellitus in the context of chronic pancreatitis, pancreatic ductal adenocarcinoma, haemochromatosis, cystic fibrosis, or prior pancreatic surgery is classified as type 3c diabetes, a form often misdiagnosed as type 2 diabetes [8, 9]. These conditions may impair beta-cell function or contribute to insulin resistance through inflammation, fibrosis, or direct disruption of pancreatic architecture. The incidence of diabetes mellitus is also elevated in patients with pNET [10, 11]. More aggressive or invasive IPMN subtypes appear more frequently in patients with diabetes, though the underlying mechanisms are still not fully understood.

Chronic systemic inflammation is a well-established contributor to impaired glucose regulation. Inflammatory conditions such as rheumatoid arthritis, as well as steroid therapy often used in their management, can exacerbate insulin resistance and complicate glycemic control. Corticosteroids elevate blood glucose levels, frequently requiring adjustments in diabetes management.

CHIP has recently gained recognition as a risk factor for age-related diseases, including T2D. In a large longitudinal study, individuals with CHIP had a 23% increased risk of developing T2D over nearly a decade of follow-up [6]. Supporting data from a Korean cohort also showed increased T2D incidence among older adults with high variant allele frequency (VAF >10%), though the study did not fully adjust for traditional risk factors [12]. Studies in mice suggest that *TET2* pathogenic variant in hematopoietic cells worsen insulin resistance, likely via increased IL-1β expression and chronic inflammation in adipose tissue. CHIP driven inflammatory pathways may therefore contribute to metabolic dysregulation, offering a link between clonal expansion and impaired glucose homeostasis. Recognizing CHIP as a contributor to glycemic instability may provide new insights into the pathogenesis of T2D in patients with complex comorbidities.

This case demonstrates how pancreatic cystic pathology, clonal hematopoiesis, and a history of autoimmune disease may contribute to atypical glucose dysregulation. Given the absence of anemia, hemoglobinopathies, renal or liver dysfunction, and with normal electrophoresis, the HbA1c discordance is most consistent with a reduced erythrocyte lifespan. The patient's chronic inflammatory states including rheumatoid arthritis, elevated ferritin, and clonal hematopoiesis of indeterminate potential likely accelerated red blood cell turnover, leading to low HbA1c values.

Elevated ferritin is a marker of iron sequestration within macrophages, a hallmark of anemia of inflammation, but even in the absence of overt anemia, inflammation can reduce red blood cell survival by 20-30% or more, as shown in both clinical and experimental studies [13].

In addition a normal albumin level does not ensure the accuracy of fructosamine, as systemic inflammation and increased protein turnover can shorten albumin's lifespan, reducing the time available for glycation. This may explain why the fructosamine-derived estimated HbA1c of 6.1% underestimated the patient's true glycemic burden despite documented hyperglycemia on CGM.

Continuous glucose monitoring provided meaningful insight into this patient's glycemia by capturing hyperglycemic episodes that were not reflected in her HbA1c. CGM remains useful in cases with suspected A1c discordance, despite limitations such as sensor accuracy and reliance on patient adherence.

## Learning points

- Hemoglobin A1c may not reliably reflect glycemic status in patients with glycemic variability, systemic inflammation, or altered red blood cell turnover.
- CGM and OGTT are essential tools for detecting postprandial hyperglycemia when HbA1c appears normal.
- Pancreatic cystic lesions, such as pNETs or IPMNs, can impair glucose regulation even without overt diabetes and should prompt metabolic evaluation.
- CHIP may contribute to inflammation-related insulin resistance or metabolic dysfunction.

## Data Availability

Original data generated and analyzed for this case report are included in this published article.
